# Identification of Potential Biomarkers for Coronary Artery Disease Based on Cuproptosis

**DOI:** 10.1155/2023/5996144

**Published:** 2023-01-25

**Authors:** Bohong Zhang, Mingliang He

**Affiliations:** ^1^Department of Anesthesiology, The Seventh Affiliated Hospital, Sun Yat-Sen University, Shenzhen, China; ^2^Department of Neurosurgery, Sun Yat-Sen Memorial Hospital, Sun Yat-Sen University, Guangzhou, China

## Abstract

Identifying peripheral biomarkers is an important noninvasive diagnosis method for coronary artery disease (CAD) which has aroused the strong interest of researchers. Cuproptosis, a newly reported kind of programmed cell death, is closely related to mitochondrial respiration, adenosine triphosphate (ATP) production, and the TCA cycle. Currently, no studies have been published about the effects of cuproptosis-related genes (CRGs) on diagnosing CAD. To screen marker genes for CAD from CRGs, we downloaded the whole blood cell gene expression profile of CAD patients and normal samples, i.e., the GSE20680 dataset, from the GEO database. By differential expression analysis, we obtained 10 differentially expressed CRGs (DE-CRGs), which were associated with copper ion response, immune response, and material metabolism. Based on the 10 DE-CRGs, we furtherly performed LASSO analysis and SVM-RFE analysis and identified 5 DE-CRGs as marker genes, including F5, MT4, RNF7, S100A12, and SORD, which had an excellent diagnostic performance. Moreover, the expression of the marker genes was validated in the GSE20681 and GSE42148 datasets, and consistent results were obtained. In mechanism, we conducted gene set enrichment analyses (GSEA) based on the marker genes, and the results implied that they might participate in the regulation of immune response. Therefore, we calculated the relative contents of 22 kinds of immune cells in CAD and normal samples using the CIBERSORT algorithm, followed by differential analysis and correlation analysis of the immune microenvironment, and found that regulatory T cell (Treg) significantly decreased and was negatively correlated with marker gene S100A12. To further reveal the regulation mechanisms, a lncRNA-miRNA-mRNA ceRNA network based on the marker genes was established. Finally, 13 potential therapeutic drugs targeting 2 marker genes (S100A12 and F5) were identified using the Drug Gene Interaction Database (DGIdb). In summary, our findings indicated that some CRGs may be diagnostic biomarkers and treatment targets for CAD and provided new ideas for further scientific research.

## 1. Introduction

Currently, coronary artery disease (CAD) is still the leading cause of death, resulting in nearly 20 million deaths globally every year, causing great harm to people's life and health and bringing a heavy burden to society [1, 2]. Unfortunately, COVID-19 is making the situation even worse. CAD is an inflammatory disease caused by atherosclerosis. Its pathogenesis involves vascular endothelial cell dysfunction, lipid deposition, macrophage activation, vascular smooth muscle cell proliferation, and migration [3]. Although considerable advance has been made in diagnosing CAD in the past 20 years, it is still urgent to explore better diagnostic markers to guide clinical treatment better and improve clinical prognosis.

Cuproptosis, a novel kind of programmed cell death, was reported by Tsvetkov et al. in the Journal of Science in March 2022. Different from the known apoptosis, pyroptosis, and ferroptosis, studies have indicated that, in the process of cuproptosis, Cu^2+^ combines with the lipoylated components of the tricarboxylic acid cycle in the mitochondrial respiratory chain, resulting in the aggregation of lipoylated protein and downregulation of iron-sulfur cluster protein, followed by proteotoxic stress as well as cell death [4]. In addition, the researchers preliminarily identified some cuproptosis-related genes (CRGs) [4]. In the past six months, diagnostic and prognostic biomarkers based on CRGs have been published in some tumors [5–7]. Previous studies have pointed out that abnormal copper levels in the body may induce a series of heart diseases, such as ischemic heart disease, arrhythmia, and heart hypertrophy. The mechanisms may be related to abnormal serum lipid metabolism caused by abnormal serum copper levels [8]. A recent study reported that a higher level of serum copper promotes the formation of atherosclerotic plaque by regulating lipid metabolism, low-density lipoprotein oxidation, and inflammatory response, thus increasing the risk of atherosclerotic heart disease [9]. Therefore, cuproptosis, copper ion-dependent programmed cell death, may be involved in the pathogenesis of CAD, and the CRGs could serve as marker genes for CAD.

In the present study, we obtained 10 differentially expressed CRGs (DE-CRGs) between CAD patients and normal samples by analyzing the GSE20680 dataset and further screened out 5 disease-signature genes, including F5, MT4, RNF7, S100A12, and SORD, by combining LASSO analysis with SVM-RFE analysis, and validated in the GSE20681 and GSE42148 datasets. According to the results of functional enrichment analyses, we preliminarily explored the relationship between marker genes and the immune microenvironment. Moreover, a ceRNA network was constructed according to the marker genes to reveal the complicated regulation mechanism. Finally, the prediction of some gene-targeted drugs was performed using DGIdb. This research may provide a novel insight into the diagnosis of CAD.

## 2. Materials and Methods

### 2.1. Data Collection

Gene expression profiles of CAD and normal samples were downloaded from the GEO database. The GSE20680 dataset (https://www.ncbi.nlm.nih.gov/geo/query/acc.cgi?acc=GSE20680), as a training set, has 139 samples, including 87 CAD patients and 52 normal samples. All patients in the cohort had signed informed consent, and the study had obtained approval from the ethics committee of Duke University. This cohort was derived from a single-center retrospective research. Inclusion criteria for the CAD patients are as follows: (1) patients with ≥70% stenosis in >1 major vessel and (2) patients with ≥50% stenosis in >2 arteries. Exclusion criterion are as follows: patients with luminal stenosis > 25% but less than 50%. The attrition number of samples is 56. Demographics of age, sex, and weight of the 139 subjects are not available. Information about randomization of subjects, blinding of investigators, power analysis for group size, and replication is not provided. The GSE20681 dataset (https://www.ncbi.nlm.nih.gov/geo/query/acc.cgi?acc=GSE20681), as a testing set, has a total of 198 samples, including 99 CAD patients and 99 normal samples. The GSE42148 dataset (https://www.ncbi.nlm.nih.go-v/geo/query/acc.cgi?acc=GSE42148), as another testing set, has a total of 24 samples, including 13 CAD patients and 11 normal samples. The original files were background adjusted and quantile normalized by the R package “limma.” There are 215 CRGs, mainly collected from literature reports and version 7.0 of the Molecular Signature Database (MsigDB) (http://www.gseamsigdb.org/gsea/msigdb/in-dex.jsp).

### 2.2. Differential Expression Analysis

From the GSE20680 dataset, we extracted the CRG expression profiles of CAD and normal samples. Using the R package “limma,” we conducted differential analyses between CAD samples and normal samples and obtained the differentially expressed genes (DEGs) with screening conditions of ∣log2(FC) | >1.0 and *p* < 0.05. The differential analyses were conducted using the Wilcoxon nonparametric test. Moreover, the results of the differential analyses were visualized using the R package “pheatmap.”

### 2.3. GO and KEGG Enrichment Analyses

Gene Ontology (GO) analysis, including biological processes, molecular function, and cellular components, is a common method to annotate genes. Kyoto Encyclopedia of Gene and Genomes (KEGG) enrichment analysis is a common method to reveal the functional information of target genes. Taking advantage of R packages “enrichplot,” “org.Hs.eg.db,” “ggplot2,” and “clusterProfiler,” we performed GO and KEGG enrichment analyses of the DE-CRGs, respectively. *p* < 0.05 and *q* < 0.05 were regarded as significantly enriched. Bar plots and bubble plots were used to visualize the results.

### 2.4. Identification and Validation of Diagnostic Genes for CAD

A candidate diagnostic gene set was screened from the DE-CRGs by the least absolute shrinkage and selection operation (LASSO) analysis using the R package “glmnet.” LASSO is a regression analysis algorithm that uses regularization to improve prediction accuracy. The penalty parameter (*λ*) of the LASSO regression model was determined by following a 10-fold cross-validation of the minimum criterion (i.e., the value of *λ* corresponding to the lowest partial likelihood deviation) [10]. Furthermore, another candidate diagnostic gene set was screened from the DE-CRGs by the support vector machine-recursive feature elimination (SVM-RFE) analysis using the R package “e1071.” SVM-RFE is an effective feature selection technique that finds the best variables by deleting the feature vector generated by SVM [11]. In this study, the SVM-RFE algorithm screened the best variables based on a minimum 10x CV error value. Using the R package “VennDiagram,” an intersection of the two gene sets was made, and the diagnostic gene set was obtained. According to the expression profile of the diagnostic gene set, the receiver operating characteristic (ROC) curves of the diagnostic genes were generated using the R package “pROC.” Furtherly, based on the diagnostic gene set, a logistic regression model was established, and the ROC curve of this model was generated. The area under the ROC curve (AUC) was obtained to judge the diagnostic efficiency. In addition, the expression levels of diagnostic genes were verified in the GSE20681 and GSE42148 datasets.

### 2.5. Gene Set Enrichment Analysis (GSEA)

“c2.cp.kegg.v2022.1.Hs.symbols.gmt” dataset as the annotation gene set was downloaded from MsigDB, and GSEA software of version 4.3.2 (https://www.gsea-msigdb.org/gsea/index.jsp) was used for analysis. Based on the cutoff value of the expression levels of marker genes, CAD samples were classified into high- and low-expression groups. The enriched pathways were sequenced using a false discovery rate (FDR) and normalized enrichment scores (NES). Moreover, *p* < 0.05 and FDR < 0.05 were considered significantly enriched.

### 2.6. Immune Infiltration Analysis

Taking advantage of the CIBERSORT algorithm, the relative contents of 22 types of infiltrating immune cells were calculated. Using the Wilcoxon rank sum test, the contents of 22 immune cells were compared between the CAD and normal samples. In addition, Spearman's correlation analyses between the expression levels of marker genes, and the contents of 22 immune cells were conducted. Using “vioplot” and “ggplot2” R packages, the results were visualized in a violin diagram and correlation heat map, respectively.

### 2.7. Construction of the ceRNA Network

lncRNA could regulate the expression of mRNA through competitively binding with miRNA. The miRNAs that targeted mRNAs of marker genes were predicted by miRanda, TargetScan, and miRDB, respectively, and only the miRNAs predicted by all three software were collected. According to the spongeScan database (https://spongescan.rc.ufl.edu), lncRNAs that interact with the above-collected miRNAs are obtained. Combining the miRNA-mRNA interactions with lncRNA-miRNA interactions, the lncRNA-miRNA-mRNA ceRNA regulatory network was obtained, and Cytoscape 3.9.1 software (https://cytoscape.org/, RRID: SCR_003032) was used to visualize the result.

### 2.8. Drug Gene Interaction Analysis of Hub Genes

The potential predictive genes were supposed as the promising drug targets for searching drugs through the Drug Gene Interaction Database (DGIdb, version 4.2.0-sha1 afd9f30b, https://dgidb.genome.wust-l.edu). The DGIdb consists of the drug gene interaction data from the DrugBank, ChEMBL, NCBI Entrez, Ensembl, PharmGKB, PubChem, clinical trials, and literature in PubMed, which can help researchers mine existing data and generate assumptions about how genes may be targeted therapeutically or prioritized for drug development. The Cytoscape (version 3.9.1) was applied to perform the drug gene interaction network.

### 2.9. Statistical Analysis

R of version 4.2.2 was used to conduct most statistical analyses in this study. In this study, variables are mainly continuous variables with nonnormal distribution, differential analysis was performed by the Wilcoxon rank sum test, and correlation analysis was performed by the Spearman correlation coefficient. For continuous variables with normal distribution, differential analysis was performed by Student's *T* test, and correlation analysis was performed by the Pearson correlation coefficient. In this study, all *p* values were bilateral, and *p* < 0.05 was considered statistical significance.

## 3. Results

### 3.1. Identification of 10 DE-CRGs in the GSE20680 Cohort

**Figure 1:**
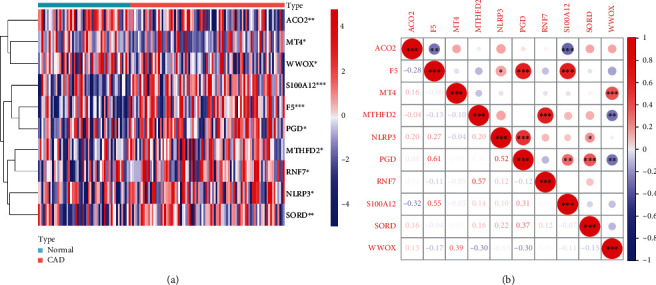
Identified 10 DE-CRGs in the GSE20680 cohort by differential expression analysis. (a) Heat map of 10 DE-CRGs in CAD patients and normal samples. Red represents high expression while blue represents low expression. (b) Spearman's correlation analyses between every two DE-CRGs. ^∗^*p* < 0.05, ^∗∗^*p* < 0.01, and ^∗∗∗^*p* < 0.001 vs. the normal group.

Using the R package “limma,” we obtained the expression profile of 199 CRGs in the GSE20680 dataset. By differential expression analysis, 10 DE-CRGs between CAD patients and normal samples were identified. Among the 10 DE-CRGs, 7 genes were upregulated, including F5, MTHFD2, NLRP3, PGD, RNF7, S100A12, and SORD, and 3 genes were downregulated, including ACO2, MT4, and WWOX (Table 1). The heat map illustrated the expression distribution of the 10 DE-CRGs in CAD patients and normal samples (Figure 1(a)). In Figure 1(b), correlation analyses between every two DE-CRGs showed significant correlations among some DE-CRGs, F5 was positively correlated with PGD and S100A12, RNF7 was positively correlated with MTHFD2, and NLRP3 was positively correlated with PGD.

### 3.2. Functional Enrichment Analyses of the 10 DE-CRGs

**Figure 2:**
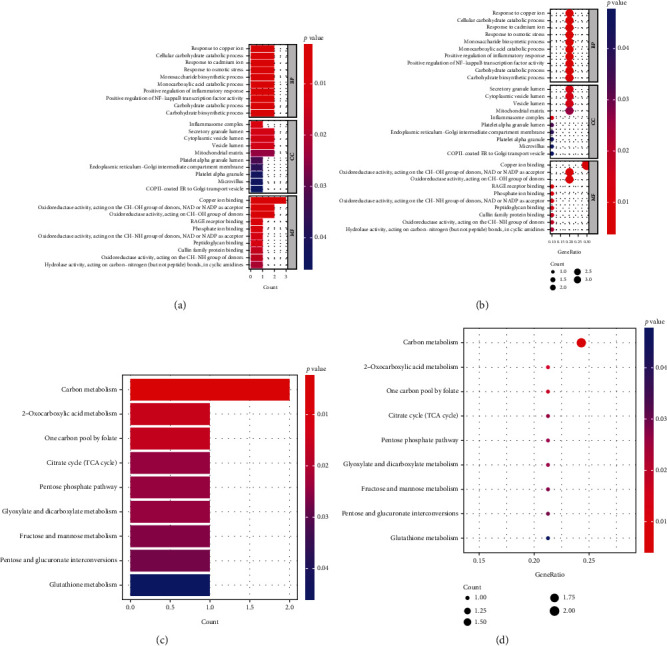
Functional enrichment analyses of the 10 DE-CRGs. (a, b) Bar plot and bubble plot of GO enrichment analysis of DE-CRGs. Top 10 GO terms enrichment in biological process (BP), cell composition (CC), and molecular function (MF). (c, d) Bar plot and bubble plot of KEGG enrichment analysis of DE-CRGs. The bar color indicates the enrichment level of DE-CRGs. Bubble size represents the number of enriched DE-CRGs, and bubble color represents the enrichment significance of DE-CRGs. *p* < 0.05 and *q* < 0.05 were considered significantly enriched.

To detect the molecular biological functions of the DE-CRGs, GO analysis and KEGG analysis were conducted. As shown in the bar plot of GO analysis (Figure 2(a)), DE-CRGs were mainly enriched in GO items related to copper ion response, copper ion binding, cellular carbohydrate catabolic process, and positive regulation of inflammatory response. In addition, as shown in the bar plot of KEGG analysis (Figure 2(b)), DE-CRGs were significantly enriched in signaling pathways related to the citrate cycle (tricarboxylic acid (TCA) cycle), 2-oxocarboxylic acid metabolism, and carbon metabolism.

### 3.3. Five DE-CRGs Were Identified as Marker Genes for CAD

**Figure 3:**
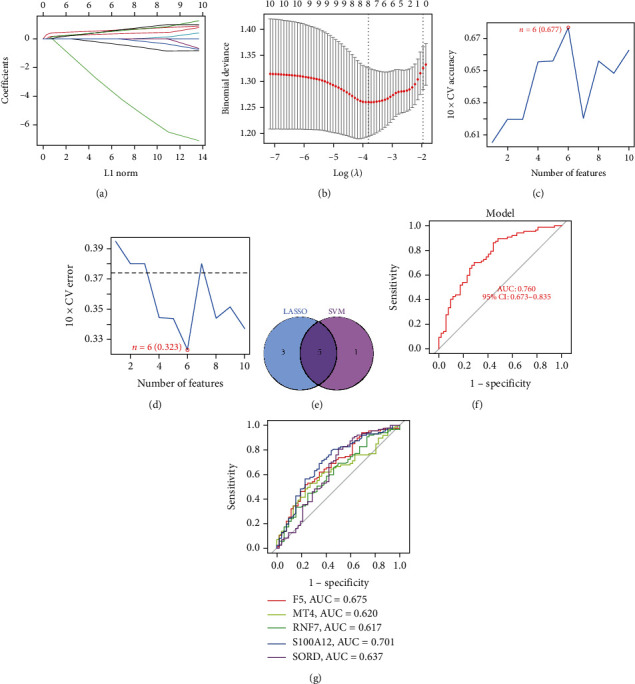
Identified 5 DE-CRGs as marker genes for CAD. (a, b) Eight DE-CRGs were identified as candidate marker genes by LASSO analysis. (c, d) Six DE-CRGs were identified as candidate marker genes by SVM-RFE analysis. (e) Venn diagram of intersection analysis. (f) ROC curve and corresponding AUC value for the logistic regression model. (g) ROC curves and corresponding AUC values for the 5 marker genes.

To identify the marker genes for CAD from the 10 DE-CRGs, we combined LASSO analysis with SVM-RFE analysis. Results of the LASSO analysis suggested that eight DE-CRGs could be served as candidate marker genes, including ACO2, F5, MT4, NLRP3, RNF7, S100A12, SORD, and WWOX (Figures 3(a) and 3(b)). In Figures 3(c) and 3(d), the results of the SVM-RFE analysis suggested that six DE-CRGs could be served as candidate marker genes, including S100A12, SORD, F5, RNF7, PGD, and MT4. In the Venn diagram, there were 5 overlapping DE-CRGs, including F5, MT4, RNF7, S100A12, and SORD; thus, they are potential marker genes for CAD patients (Figure 3(e)). Moreover, a logistic regression model was established according to the 5 marker genes. To determine the diagnostic performance, ROC curves of each gene and the model were generated, respectively. Results indicated that the AUC value of the model was 0.760 (Figure 3(f)), and the AUC values of F5, MT4, RNF7, S100A12, and SORD were 0.675, 0.620, 0.617, 0.701, and 0.637, respectively (Figure 3(g)).

### 3.4. Marker Genes of CAD Were Correlated with Immune Regulation

**Figure 4:**
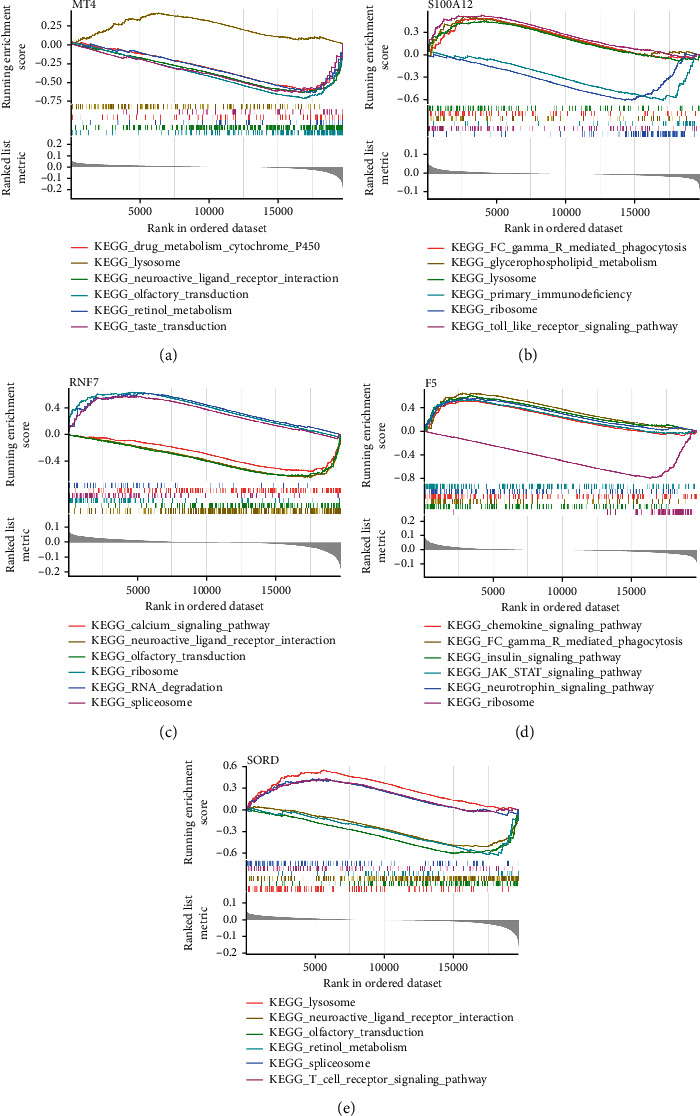
Marker genes of CAD were correlated with immune regulation. (a) GSEA of marker gene MT4. (b) GSEA of marker gene S100A12. (c) GSEA of marker gene RNF7. (d) GSEA of marker gene F5. (e) GSEA of marker gene SORD.

To further study the molecular biological functions of the marker genes, GSEA was conducted. As shown in Figures 4(a)–4(e), results of GSEA of each marker gene showed that marker genes were mainly enriched in the metabolism (retinol metabolism, drug metabolism cytochrome p450, and glycerophospholipid metabolism), immune response (T cell receptor signaling pathway and chemokine signaling pathway), JAK-STAT signaling pathway, Toll-like receptor signaling pathway, insulin signaling pathway, ribosome, and lysosome.

### 3.5. Immune Microenvironment Analysis

**Figure 5:**
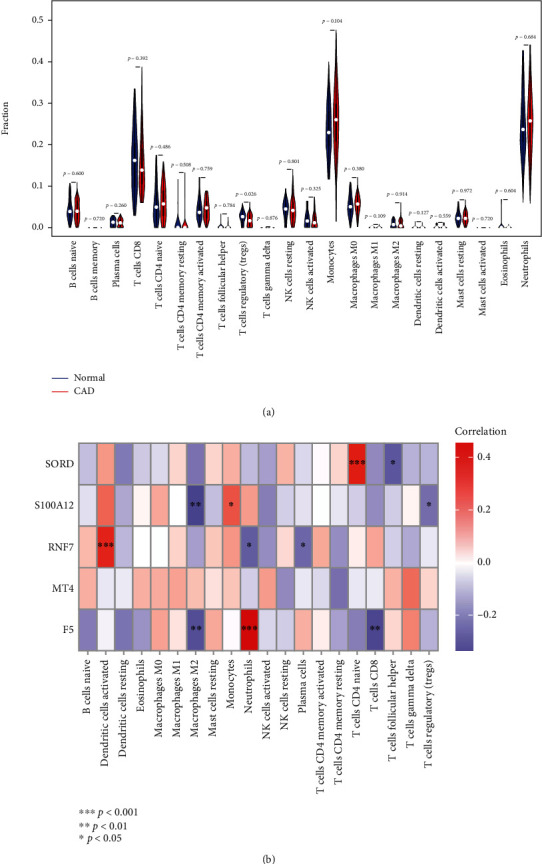
Immune microenvironment analysis. (a) By the Wilcoxon rank sum test, a differential analysis of immune cell contents between CAD samples (red) and normal samples (blue) was conducted, and the results were shown in a violin diagram. (b) Correlation heat map of Spearman's correlation analysis between 22 immune cells and marker genes.

To evaluate the composition of immune cells in the microenvironment, the relative contents of 22 immune cells in every CAD and normal sample were calculated using the CIBERSORT algorithm. To verify immune microenvironment participates in the pathogenesis of CAD, we conducted differential analyses of immune cell contents between CAD samples and normal samples, followed by correlation analyses between marker genes and 22 immune cells. Results of differential analyses showed that the relative content of Tregs in CAD samples was lower than that in the normal samples (Figure 5(a)). Moreover, the results of correlation analyses showed that the relative content of Tregs was negatively correlated with the expression level of S100A12 (*p* < 0.05) (Figure 5(b)). Thus, S100A12 might participate in the pathogenesis of CAD by regulating Tregs.

### 3.6. Established a lncRNA-miRNA-mRNA ceRNA Network according to the Marker Genes

**Figure 6:**
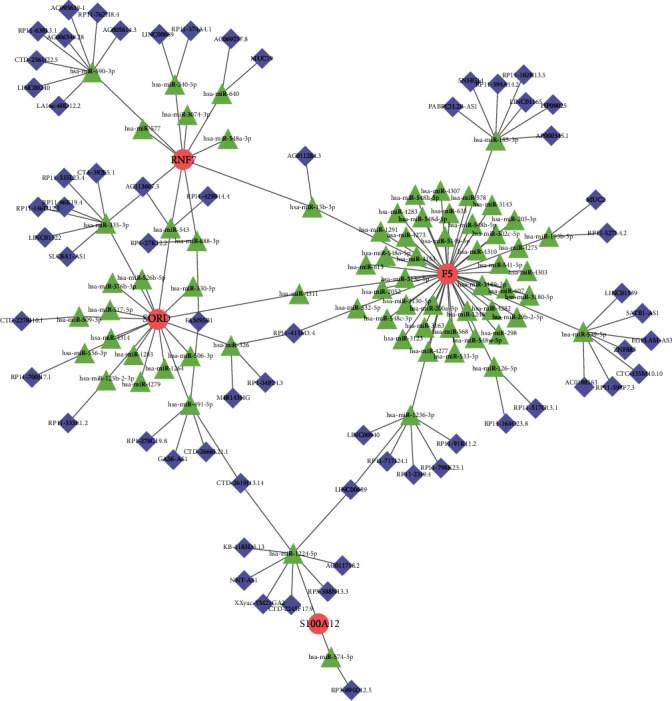
Established a lncRNA-miRNA-mRNA ceRNA network based on marker genes. The complex network included 139 nodes (64 lncRNAs, 71 miRNAs, and 4 marker genes) and 143 edges. Blue diamond represents lncRNAs, green triangle represents miRNAs, and red ellipse represents mRNAs.

To further reveal the regulation mechanisms, a lncRNA-miRNA-mRNA regulatory network was established. The complex network included 139 nodes (64 lncRNAs, 71 miRNAs, and 4 marker genes) and 143 edges. As shown in Figure 6, the ceRNA network was roughly divided into four clusters connected by several miRNAs. F5 was located in the center of a cluster and was directly connected by 46 miRNAs, in which hsa-miR-185-3p, hsa-miR-539-5p, and hsa-miR-1236-3p were competitively bound by multiple lncRNAs, respectively. SORD was located in the center of another cluster and was directly connected by 17 miRNAs, in which hsa-miR-335-3p, hsa-miR-326, and hsa-miR-491-5p were competitively bound by multiple lncRNAs, respectively. For S100A12, S100A12 was regulated by 2 miRNAs, including hsa-miR-1224-5p and hsa-miR-574-5p, and the 2 miRNAs were competitively bound with a total of 9 lncRNAs. These results revealed that these marker genes might play core roles in CAD.

### 3.7. Prediction of Targeted Drugs or Compounds

**Figure 7:**
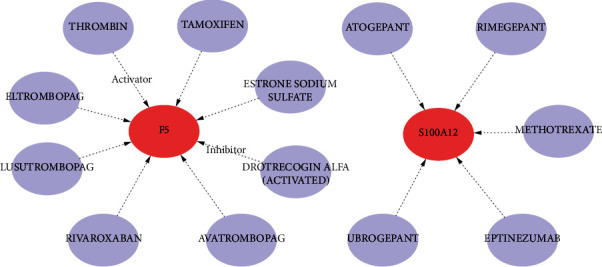
Prediction of targeted drugs or compounds. By DGIdb, 13 drugs or compounds targeting 2 marker genes (F5 and S100A12) were identified. The drug gene interaction network contained 15 nodes and 13 sides. Red nodes represent genes, and blue nodes represent the drugs or compounds. The lines represent the interaction relationship between the genes and the drugs or compounds.

To preliminarily explore the treatment for CAD, potential gene-targeted drugs were predicted using the DGIdb database. As shown in Figure 7, 13 drugs or compounds targeting 2 marker genes were identified. Among the 13 drugs or compounds mentioned above, thrombin is an activator for F5, and drotrecogin alfa (activated) is an inhibitor for F5. As for marker gene S100A12, there are 5 candidate gene-targeted drugs or compounds, including atogepant, rimegepant, methotrexate, eptinezumab, and ubrogepant (Figure 7). However, we did not identify gene-targeted drugs or compounds for SORD, RNF7, and MT4.

### 3.8. Validation of the Expression Levels of the Marker Genes

**Figure 8:**
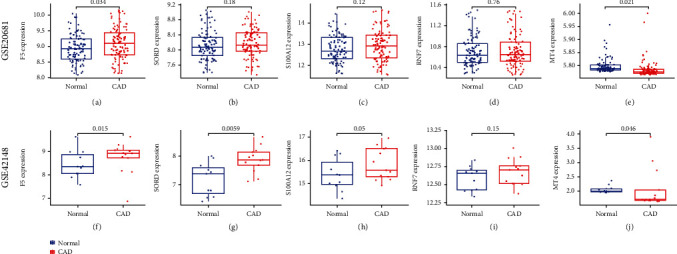
Validated the expression levels of the 5 marker genes in two testing sets. (a–e) Differential expression analyses of F5, SORD, S100A12, RNF7, and MT4 between CAD samples and healthy controls in the GSE20681 dataset. (f–j) Differential expression analyses of F5, SORD, S100A12, RNF7, and MT4 between CAD samples and healthy controls in the GSE42148 dataset.

To validate the expression levels of marker genes, differential expression analyses of the 5 marker genes between CAD patients and normal samples were performed in the GSE20681 dataset and GSE42148 dataset, respectively. Consistently, in the GSE20681 dataset, the expression level of F5 in CAD patients was higher than that in normal samples (*p* = 0.034), while the expression level of MT4 in CAD patients was lower than that in normal samples (*p* = 0.021). Moreover, although it did not reach statistical significance, the expression levels of RNF7, S100A12, and SORD in CAD samples tended upregulation (Figures 8(a)–8(e)). Similarly, in the GSE42148 dataset, the expression levels of F5, SORD, and S100A12 in CAD patients were significantly higher than in normal samples. The expression level of RNF7 in CAD patients tended to upregulation, while the expression level of MT4 in CAD patients was significantly lower than that in normal samples (Figures 8(f)–8(j)).

## 4. Discussion

CAD is a heart disease caused by atherosclerosis, and atherosclerosis is a progressive inflammatory disease [12]. The endothelial cell dysfunction initiates atherosclerosis' pathological process [13]. Then, the macrophages abnormally aggregate and release a lot of cytokines [14]. CAD is a chronic disease and may last for decades. Currently, radioangiography is the gold standard for the diagnosis of CAD [15]. However, this technique is not suitable for routine use. Therefore, the identification of novel markers for early diagnosis is urgently needed. In recent years, according to the gene expression profiles obtained from public databases, researchers have identified various diagnostic biomarkers in CAD through different bioinformatic analysis methods.

Among these biomarkers, the genes associated with cell death are important. Research indicated that apoptosis, pyroptosis, parthanatos, and autophagy could regulate the risk of CAD [16]. Additionally, necroptosis and ferroptosis are related to the pathogenesis of CAD [16]. Ferroptosis, iron-dependent programmed cell death, is featured lipid peroxidation of unsaturated fatty acids via Fe^2+^, ultimately leads to cell death [17]. Ding et al. constructed and validated a reliable diagnostic model for CAD based on the expression profiles of 16 ferroptosis-related genes, including ZFP36, VDAC2, TNFAIP3, SCP2, RPL8, PIK3CA, PCBP1, MTDH, MIF, MAP1LC3B, HIF1A, FTH1, CASP8, BACH1, ATP5MC3, and ACSL1, and its AUC value was 0.971 [18]. Similarly, Wu et al. identified 7 ferroptosis-related genes as biomarkers for CAD via bioinformatics analysis, including TRIB3, STMN1, SLC1A4, HSPB1, CEBPG, CBS, and CA9, and its AUC value in the training set was 0.748 [19]. By combining ferroptosis with necroptosis, Liu et al. identified 4 ferroptosis- and necroptosis-related genes as marker genes for the diagnosis of CAD, including TLR4, CBS, LONP1, and HSPB1 [20]. However, there is no study on cuproptosis-related biomarkers in CAD.

In March 2022, Tsvetkov et al. proposed for the first time a copper ion-dependent and novel programmed cell death type, namely, cuproptosis. Research indicated that Cu2^+^ combines with the lipoylated components of the tricarboxylic acid cycle in the mitochondrial respiratory chain, resulting in the aggregation of lipoylated protein and downregulation of iron-sulfur cluster protein, followed by proteotoxic stress as well as cell death [4]. Here, we performed a differentially expressed analysis of 199 CRGs between the CAD and normal samples and obtained 10 DE-CRGs. By combining LASSO analysis with SVM-RFE analysis, we established a diagnostic signature containing 5 DE-CRGs, and its AUC value was 0.760. Though this AUC value is not the highest, it is still higher than most reported studies [19, 21, 22]. The diagnostic performance of these marker genes is superior to that of most reported biomarkers. In addition, the expression levels of marker genes were verified in the GSE20681 dataset and GSE42148 dataset, respectively, and relatively consistent results were obtained.

The diagnostic signature established in this study included the following 5 DE-CRGs: F5, MT4, RNF7, S100A12, and SORD. F5 (coagulation factor V), a circulating procofactor, participates in the process of blood coagulation [23]. A study has reported that 1628 G → A polymorphism of F5 is associated with CAD and may be a risk factor for CAD in the Chinese population [24]. Similarly, in our study, F5 is upregulated in CAD patients and serves as a marker gene for CAD. MT4 (also named MMP17), a new member of the matrix metalloproteinase family, locates at the plasma membrane [25]. A study has reported that MT4 deficiency promotes the recruitment of monocytes and thus promotes atherosclerosis [26]. Consistently, in this diagnostic signature established, MT4 serves as a protective factor and is downregulated in CAD patients. S100A12/calgranulin C, a member of the S100 calcium-binding proteins family, is mainly expressed and secreted by granulocytes [27]. Studies suggested that S100A12 could promote the occurrence and development of atherosclerosis by inducing the production of inflammatory factors [28–31]. Zhao et al. reported that the serum level of S100A12 is independently related to the risk of CAD [30]. Consistently, our results showed that S100A12 is significantly upregulated in CAD patients and may serve as a marker gene for CAD. RNF7, also known as SAG, was initially identified as an antioxidant protein. RNF7 is also a component of E3 ubiquitin ligases [32]. SORD, a member of the dehydrogenase/reductase protein family, could participate in glucose metabolism [33]. However, relatively few studies have been conducted on RNF7 and SORD, and their relationship with CAD is poorly understood. The biological functions of most marker genes are consistent with that reported previously, which also reflects the reliability of our results.

CAD is caused by coronary atherosclerosis, a progressive inflammatory disease [34]. Researchers pointed out that the immune microenvironment plays an important role in the initiation and progression of CAD [35]. Regulatory T cells are a unique T cell subset known as Foxp3^+^CD25^high^CD4^+^CD127^low^ Treg cells. Tregs weaken immune responses by inhibiting the proliferation of other T cells. Many studies have indicated that Tregs could inhibit the occurrence of atherogenesis [36–40]. Emoto et al. reported that the relative content of Tregs was lower in CAD samples than that in healthy people [41]. Wang et al. also reported a significant decrease in the absolute number of Tregs in rheumatoid arthritis patients with CAD (RA-CAD group) compared to the pure RA group [42]. Consistently, our research indicated that the relative content of Tregs was lower in CAD patients compared with that in normal samples. Therefore, it might be a promising therapeutic approach for CAD to promote Treg response. Moreover, results of GSEA indicated that marker genes were also enriched in metabolism, such as glycerophospholipid metabolism. Consistently, Chen et al. reported that glycerophospholipid metabolism appeared to be a predominant alteration in CAD progression by metabolomics analysis [43]. Abnormal lipid metabolism and immune inflammation have been proven to participate in the development and progression of CAD. Abnormal blood lipid metabolism, especially low-density lipoprotein (LDL), is an important risk factor for CAD [12]. In addition, Kunutsor et al. reported that a higher level of serum copper promotes the formation of atherosclerotic plaque by regulating lipid metabolism and LDL oxidation, thus increasing the risk of atherosclerotic heart disease [9]. Therefore, cuproptosis-related genes might participate in the pathogenesis of CAD by regulating lipid metabolism.

Finally, we preliminarily explored the potential regulatory mechanisms and predicted some therapeutic targets and drugs. However, these are still in the prediction stage and need further verification by experiments.

## 5. Conclusions

In this research, we obtained 10 DE-CRGs between CAD patients and normal samples by analyzing the gene expression profiles of the GSE20680 dataset, followed by combining LASSO analysis with SVM-RFE analysis, and identified 5 DE-CRGs as marker genes, including F5, MT4, RNF7, S100A12, and SORD. Then, we validated their expression levels in the GSE20681 and GSE42148 datasets. After GO, KEGG, and GSEA analyses, we conducted a differential analysis of the immune microenvironment between CAD patients and healthy people, followed by correlation analysis between marker genes and 22 immune cells, and results indicated that Treg is the main alteration in the immune microenvironment which may be related to marker gene S100A12. Moreover, we constructed a ceRNA network and predicted the gene-targeted drugs. This study may provide a novel insight into the early diagnosis of CAD.

## Figures and Tables

**Table 1:** Ten of 199 CRGs were differentially expressed between CAD and normal samples, including 7 upregulated and 3 downregulated genes.

Gene	*p* value	Expressing trend
ACO2	0.009907	DN
F5	0.000575	Up
MT4	0.017889	DN
MTHFD2	0.029526	Up
NLRP3	0.039715	Up
PGD	0.02555	Up
RNF7	0.021669	Up
S100A12	7.33E-05	Up
SORD	0.006961	Up
WWOX	0.045487	DN

DN: downregulated; Up: upregulated.

## Data Availability

The GSE20680, GSE20681, and GSE42148 datasets were downloaded from the GEO database.
